# Nanofibrous Polycaprolactone Membrane with Bioactive Glass and Atorvastatin for Wound Healing: Preparation and Characterization

**DOI:** 10.3390/pharmaceutics15071990

**Published:** 2023-07-20

**Authors:** Mohamed S. El-Okaily, Amany A. Mostafa, Judyta Dulnik, Piotr Denis, Paweł Sajkiewicz, Azza A. Mahmoud, Reham Dawood, Amr Maged

**Affiliations:** 1 Refractories, Ceramics and Building Materials Department (Biomaterials Group), National Research Centre (NRC), El Bohouth St., Dokki, Giza 12622, Egypt; ms.ibrahim1987@gmail.com; 2 Nanomedicine & Tissue Engineering Lab., Medical Research Center of Excellence (MRCE), National Research Centre (NRC), Giza 12622, Egypt; 3 Laboratory of Polymers and Biomaterials, Institute of Fundamental Technological Research Polish Academy of Sciences, Pawińskiego 5b, 02-106 Warsaw, Poland; jdulnik@ippt.pan.pl (J.D.); pdenis@ippt.pan.pl (P.D.); psajk@ippt.pan.pl (P.S.); 4 Pharmaceutics and Pharmaceutical Technology Department, Faculty of Pharmacy, Future University in Egypt, New Cairo 11835, Egypt; amr.maged@fue.edu.eg; 5 Microbial Biotechnology Department, Biotechnology Research Institute, National Research Centre, EL Bohouth St., Dokki, Giza 12622, Egypt; rmhaemd@hotmail.com; 6 Pharmaceutical Factory, Faculty of Pharmacy, Future University in Egypt, New Cairo 11835, Egypt

**Keywords:** nanofibers, polycaprolactone, bioactive glass, coating, wound healing

## Abstract

Skin wound healing is one of the most challenging processes for skin reconstruction, especially after severe injuries. In our study, nanofiber membranes were prepared for wound healing using an electrospinning process, where the prepared nanofibers were made of different weight ratios of polycaprolactone and bioactive glass that can induce the growth of new tissue. The membranes showed smooth and uniform nanofibers with an average diameter of 118 nm. FTIR and XRD results indicated no chemical interactions of polycaprolactone and bioactive glass and an increase in polycaprolactone crystallinity by the incorporation of bioactive glass nanoparticles. Nanofibers containing 5% *w/w* of bioactive glass were selected to be loaded with atorvastatin, considering their best mechanical properties compared to the other prepared nanofibers (3, 10, and 20% *w/w* bioactive glass). Atorvastatin can speed up the tissue healing process, and it was loaded into the selected nanofibers using a dip-coating technique with ethyl cellulose as a coating polymer. The study of the in vitro drug release found that atorvastatin-loaded nanofibers with a 10% coating polymer revealed gradual drug release compared to the non-coated nanofibers and nanofibers coated with 5% ethyl cellulose. Integration of atorvastatin and bioactive glass with polycaprolactone nanofibers showed superior wound closure results in the human skin fibroblast cell line. The results from this study highlight the ability of polycaprolactone-bioactive glass-based fibers loaded with atorvastatin to stimulate skin wound healing.

## 1. Introduction

Skin defects or cell abnormalities resulting from a physical, thermal or underlying illness are referred to as wounds [1]. A natural biological response to tissue damage is wound healing. The usual wound healing cycle typically lasts between four and six weeks (an acute wound). Wounds that take longer than this to heal are referred to as chronic wounds [2]. Patients and medical professionals all over the world face difficulties because of chronic wounds. These wounds are linked to high death rates and significantly negatively influence life quality [3].

Hypoxia, bacterial colonization, ischemia, reperfusion damage, an impaired cellular reaction, and defects in collagen production are some of the underlying factors that result in chronic wounds. They may be brought on by chronic conditions, such as malnutrition or systemic disease like diabetes [4,5]. Diabetes-related foot ulcers are particularly deadly, with fatality rates that exceed those of several common malignancies [6]. Individuals with other chronic wounds, such as pressure ulcers and venous leg ulcers, also show a greater mortality rate than patients of similar age [7].

In order to treat these wounds, wound dressings are essential because they allow for airflow, shield the wound from the outside environment, and provide an appropriate moist environment that prevents wound desiccation while allowing for exudate absorption [4,8]. Traditional dressings have not been able to keep up with the need for the care and treatment of wounds due to an increase in demand globally. Electrospun nanofiber membranes offer an architectural similarity to the fibrillar part of the natural extracellular matrix and are considered a sophisticated form of wound dressing [9].

Researchers are becoming increasingly interested in electrospinning technology as a versatile and straightforward technique for manufacturing [10,11]. Electrospinning is an electrohydrodynamic process that depends on the electrostatic repulsion between charges on the surface to generate nanofibers from a viscoelastic fluid [12]. Because of their high surface-to-volume ratio and ability to create a highly porous mesh, nanofibers perform better in many applications [13]. Nanofibers have been produced from a variety of materials using electrospinning. The most common materials used are organic polymers in solution or melt form [14]. Therefore, they may be dissolved in suitable solvents to produce solutions or melted without degrading. The majority of organic polymers can be used directly in electrospinning [14].

The electrospun nanofiber membrane has a distinctively small hole size and high porosity that can both prevent pathogen infection of the wound and guarantee the free movement of gas and liquid molecules [15,16]. Moreover, nanofiber membranes can serve as biomimetic scaffolds at the wound to stimulate the regeneration of new tissue and cell growth [10]. Also, they offer enormous promise for loading and delivering drugs that aid in wound healing [17].

Polycaprolactone (PCL) is a synthetic polyester that is non-toxic, biodegradable and has good biocompatibility [9]. This polymer has a high degree of flexibility and a moderate rate of degradation [18,19]. PCL is one of the most efficient materials for electrospinning nanofibers since it has a high level of biocompatibility. [9]. It was used to prepare nanofiber membranes for different uses, such as in biomedical applications [20,21] and as an implant [22,23]. Due to the high degree of crystallinity in PCL membranes, drug release is relatively delayed from these membranes [24].

Statins are medications used to lower lipids and prevent coronary heart disease by inhibiting HMG-CoA reductase. Atorvastatin is one of the statin medications that raise the proportion of LDL receptors on hepatic cells’ surface and reduces the amount of cholesterol produced in the liver [25]. Atorvastatin calcium is marginally soluble in distilled water and phosphate buffer with a pH of 7.4. Due to presystemic clearance in the gastrointestinal mucosa and/or hepatic first-pass metabolism, atorvastatin has a 14% absolute bioavailability and a 30% systemic availability of HMG-CoA reductase inhibitory action [26].

Statins revealed beneficial effects on wound healing due to their numerous pleiotropic actions, which can affect cellular functions like apoptosis, inflammation, and proliferation [27]. Also, they showed increased endothelial and microvascular functioning and reduced oxidative stress, all of which may speed up and enhance the healing process [28,29]. Statins, especially atorvastatin, have been shown to speed up the healing process in both human and animal trials [30]. By regulating the expression of proteins and cytokines linked to the pathways of cell growth, atorvastatin has been demonstrated to accelerate tissue repair [27]. Animals treated with bioscaffolds made of chitosan and hydroxyapatite containing atorvastatin contracted their wounds completely [31,32]. Atorvastatin nanoemulgel showed a significant improvement in the histological architecture of the skin, along with a high percentage of wound contraction [33]. The maximum amount of wound-healing power was shown by atorvastatin loaded in niosomes (non-ionic surfactants vesicles) gel, which led to full epithelization and wound closure after just 21 days [34].

Silicate-based bioactive glasses (SBGs) are a class of highly-reactive surface glass–ceramic biomaterials [32,35]. The use of wound dressing with borate-base glass has been given regulatory permission to cure both chronic and acute wounds [36,37]. SBGs have been shown to stimulate gene expression associated with the processes of healing, including vascular cell adhesion protein, vascular endothelial growth factor, and essential fibroblast growth factor for angiogenesis [38,39,40]. Moreover, it has been demonstrated that bioglass 45S5 promotes gap–junction communication and endothelial cell protection, both of which boost vascularization and speed up wound healing [41]. Moreover, it has been suggested that the rise in pH caused by the breakdown of silicate-based glasses has an antimicrobial impact [42]. Furthermore, Si^4+^ is one of the ionic components generated from SBGs that showed a substantial role in boosting the production of collagen, which is crucial in preventing the formation of scar tissue [43,44]. TGF-β (transforming growth factor-β) has a variety of impacts on immunological regulation, ECM formation, and cell differentiation and proliferation. On the other hand, pathological skin conditions such as chronic wounds and severe scarring have been linked to aberrant TGF-β signaling [44]. In fact, an in vitro study found that direct contact with a silicate-derived sol-gel (SBG) reduced the activity of the downstream molecule Smad2 and TGF-β signaling, suggesting that BGs might influence modifying the pathway of TGF-β [45].

The ability of bioactive glass nanoparticles to induce the growth of new tissue makes them ideal for wound healing [46,47]. In a previous work [48], we prepared novel SBGs by the sol–gel method, fabricated and characterized, revealing prominent potential in biomedical applications. Small molecules of bioactive glass can also be directly introduced into nanofibers during electrospinning. Additionally, in recent years, synthetic biomaterials and active pharmaceutical components have become increasingly utilized for techniques to treat wounds. The present work aimed to synthesize electrospun composite nanofiber membranes containing BG nanoparticles to analyze the properties and structure of PCL-based nanofiber membranes loaded with bioactive glass nanoparticles to the composites. 

The aim of the present work depends on the integration of the innovative sol–gel SBGs fabricated in PCL-based nanofiber membranes and on the characterization of the interaction of these bioactive glass nanoparticles with the polymeric matrix to grasp the possibility of using such material systems with atorvastatin as an effective wound healing membrane. Such membranes will be expected to serve as biomimetic scaffolds at the wound to stimulate and support cell growth for the enhancement of the wound healing process.

## 2. Materials and Methods

### 2.1. Materials

Polycaprolactone (PCL, molecular weight = 80,000 g/mol) was obtained from Sigma-Aldrich, UK. Acetic acid (AA, 99.5%) was obtained from Chempur, Poland. Formic acid (FA, 98–100%) was purchased from Avantor Performance Materials, Poland.

The sol-gel technique was applied to prepare nano-bioactive glass (SBG) with a composition of 46% Na_2_O, 24% CaO, 24% SiO_2_, and 6% P_2_O_5_ wt.% using the modified method described in previously published work and its detailed characteristics are provided in [48,49]. SBG has a particle size value of 32.67 nm and a zeta potential value of –14.0 ± 0.02 mV at pH 7.3 [48]. Atorvastatin calcium was supplied by the EIPICO Pharmaceutical Company, Egypt. Ethocel standard 100 Premium (ethyl cellulose) was obtained from Colorcon, USA. All chemical reagents were of analytical grade, obtained from commercial suppliers, and utilized without further purification.

### 2.2. Methods

#### 2.2.1. Preparation of the Electrospinning Solutions

A blend of acetic acid and formic acid (AA/FA) with a ratio of 9:1 *w/w* was utilized as the solvent. The total concentration for the solid content (PCL and SBG) dissolved in the AA/FA system was fixed at 10% *w/w*. The prepared solution was heated for 4 h at 50 ± 5 °C under continuous stirring (500 rpm) to ensure the dissolution of the PCL polymer.

Nano-powder of SBG was added to the PCL solution (C1) in four different weight percentages of 3, 5, 10 and 20% *w*/*w* (from total solid content) to produce C1-B3, C1-B5, C1-B10 and C1-B20 solutions, respectively (Table 1). The viscosities of the solutions were determined at 50 rpm using a Brookfield viscometer DV-E, U.S.A., at room temperature. The formulations were sonicated for 30 min to eliminate air bubbles before electrospinning.

#### 2.2.2. Electrospinning Process

**Figure 1:**
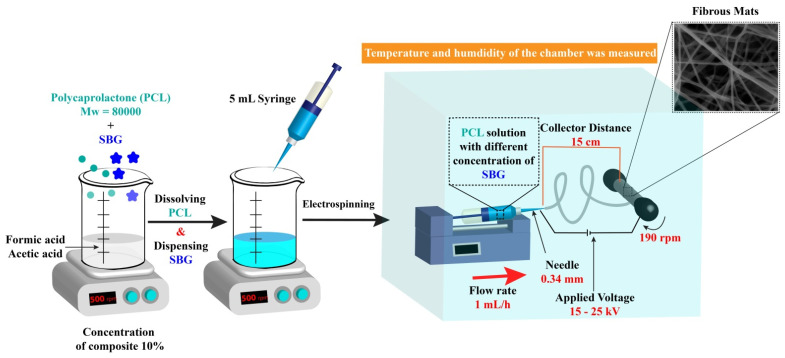
Schematic diagram for the preparation of PCL-based fibers.

The electrospinning apparatus operated horizontally (Figure 1). It comprised two pumps with syringes (New Era Pump Systems, NE-1000 model, NY, USA and KD Scientific KDS-100-CE model, Holliston, USA). The two needles were mounted on either side of a grounded spinning drum collector (190 rpm) with a radius of 4 cm and a length of 12 cm. The distance between the needles and the collector was 15 cm. The positive terminals of two high-voltage generators were linked to stainless steel needles with an inner diameter of 0.34 mm. The spinning solution’s flow rate was set at one mL/h on both sides. For successful electrospinning of the prepared solutions, a voltage between 15 and 25 kV was utilized (Table 1) to produce uniform fibers and maintain a steady electrospinning operation. The electrospinning process was adjusted at a humidity of 50–55% and a temperature of 22–25 °C. All materials were exposed to fume hoods for two days after electrospinning to ensure no solvent residue remained in the fibers before the subsequent experimental processes.

#### 2.2.3. Characterization of the Prepared Nanofibers

##### Scanning Electron Microscopy (SEM)

The produced nanofiber membrane was cut from the center to analyze the morphology of the nanofibers under the Fe-SEM Philips apparatus, U.S.A., model QUANTA FEG 250. The nanofibers were gold-sputtered at 15 mA for 3.5 min before analysis.

##### Fourier Transform Infrared (FT-IR) Spectra and X-ray Diffraction (XRD) Analysis

To identify the type of chemical bonds in the nanofibers, Fourier transform infrared (FT-IR) spectra were taken with wave numbers between 2000 and 400 cm^−1^ for the nanofibers using a Vertex 70 spectrometer (Bruker Optics, Germany) by using the ATR technique. A scan speed of 2 mms^−1^ with a spectral resolution of 2 cm^−1^ was applied.

The crystallinity of the nanofibers was investigated using a Bruker D8 (Bruker, Karlsruhe, Germany) Discover diffractometer with the CuK_α_ (wavelength 1.5418 Å) radiation at 40 mA and 40 kV (1600 W nominal power) over the ranges of 2θ = 10°–33°. Reflective mode with coupled-theta-2theta geometry and a fast 1D linear Lynxeye detector were used. Background correction was done in Bruker EVA software, and subsequent data analysis was held in Origin software (XRD profiles deconvolution using Pearson VII function). Deconvolution was controlled manually, with at least 100 correction calculations of the cumulative curve. 

The crystallinity of the PCL, X_PCL_, was determined from the ratio of the area of the analyzed crystal diffraction peaks from the PCL to the total area of scattering (including the area of amorphous peak) registered in the angular range 2θ between 10 and 33°.

##### Mechanical Tests

The mechanical characteristics of the nanofiber membranes were measured using a uniaxial testing apparatus (Lloyd EZ-50 with handles for fragile materials, UK) with a 50 N load cell and a crosshead speed of 10 mm/min. For each material type, three samples measuring 50 mm × 10 mm (with a 25 mm × 10 mm extension between handles) were examined. The thickness of the samples was measured before being used in the analysis and found to be in the range of 50 to 80 µm. Tensile strength and Young’s modulus were estimated using the stress–strain curves. 

#### 2.2.4. Loading Atorvastatin on the Nanofibers

Atorvastatin (10 mg/mL) was dissolved in 5% and 10% ethyl cellulose ethanolic solutions to be loaded into the nanofiber membranes by the dip-coating method. Selected nanofibers were quickly immersed in the ethyl cellulose–ethanolic solution for five seconds, followed by drying at room temperature. Also, for comparative purposes, atorvastatin was loaded on the nanofiber membrane without the use of ethyl cellulose.

The amount of atorvastatin loaded in the prepared nanofibers was determined by soaking the nanofibers in ethanol, and the drug concentration was measured spectrophotometrically at 240.6 nm.

#### 2.2.5. In Vitro Drug Release

The atorvastatin release in the nanofibers was determined using an incubator shaker (IKA KS-4000, Germany). In brief, nanofiber membranes (1 cm × 1 cm) were soaked in 20 mL of the release medium (phosphate buffer saline with a pH value of 7.4). The speed of the shaker was adjusted at 100 rpm and a temperature of 37 °C. Samples of 3 mL were taken at different time intervals and measured spectrophotometrically at 240.6 nm to determine the concentration of drug release at each time interval, and they were replaced with fresh media. The release efficiency (RE) [50] and mean release time (MRT) [51] were estimated using KinetDS 3.0 software (Poland).

The kinetics release of atorvastatin was estimated using the Korsmeyer–Peppas equation [52,53]:

Q_t_/Q_∞_ = kt^n^ (1)

where Q_t_/Q_∞_ is the fraction of drug released at any time t; K is the release-rate constant, and n is the diffusional exponent value, which determines the mechanism of drug release.

SEM images were taken from the soaked nanofiber membranes in the release medium after 2 h of drug release. The samples were dried and scanned to detect the spaces between nanofibers using a scanning electron microscope (Quanta™ 250 FEG, USA). 

#### 2.2.6. Ex Vivo Studies on the Human Skin Fibroblast Cell Line

The Human Skin Fibroblast (HSF) cell line was provided by Nawah Scientific Inc. (Mokatam, Cairo, Egypt). Cells were cultured at 37 °C in DMEM media supplemented with 100 mg/mL streptomycin, 100 units/mL penicillin, and 10% heat-inactivated fetal bovine serum. The incubator was humidified and contained 5% (*v*/*v*) CO_2_.

##### Cytotoxicity Assay (MTT Assay)

The MTT (3-(4, 5-dimethyl thiazol-2yl)-2, 5-diphenyl tetrazolium bromide) experiment was utilized to measure cytotoxicity. Briefly, the DMEM media with 10% FBS and 1% penicillin–streptomycin was utilized to seed cells onto 96-well dishes. Cells were subsequently grown at 37 °C for a period of 24 to 48 h in a humid environment with 5% CO_2_. Fresh media with various drug concentrations was applied to the wells when the required level of cell confluence was achieved. After the incubation period, the MTT stain was applied, and the formazan crystals were dissolved with DMSO following the re-incubation. The wells’ absorbance at 570 nm was determined utilizing a microplate reader. The results are presented as a percentage of inhibition compared to control cells, with cell survival assumed to be 100%. Every cell viability test was carried out in triplicate.

##### Wound Healing Assay

The ethical approval for this study was given by the ethics research committee, Faculty of Pharmacy, Future University in Egypt, Cairo, Egypt (REC-FPFUE-20/2023). The effects of nanofiber membranes prepared using PCL containing SBG and atorvastatin on skin fibroblasts via a wound healing assay were examined to be used as wound dressings in the future and to improve the skin repair process.

Human Skin Fibroblast (HSF) cells were seeded with a 2 × 105/well density into a 12-well dish and cultivated nightly at 37 °C and 5% CO_2_ in 5% FBS-DMEM to determine the scratch wound test [54]. The following day, the confluent monolayer (1.15 ± 0.0264 mm) was scratched horizontally. The dish was completely cleaned with PBS, and the tested nanofiber membranes were added to each treated well, whereas the untreated wells received fresh medium as a control group. At the stated time intervals, an inverted microscope was used to capture the images. The dish was incubated at 37 °C with 5% CO_2_ between periods. ImageView MII software (version 3.7) was used to analyze the captured images, which are shown below.

The gap distance between the cells (scratch) was measured for the following tested groups:

Group 1—Control group: did not receive any treatment.

Group 2—C1-B5 group: treated C1-B5 nanofiber membrane.

Group 3—C1-B5-AV group: treated C1-B5 nanofiber membrane loaded with atorvastatin.

Group 4—C1-B5-AV-10% Coat group: treated C1-B5 nanofiber membrane coated with 10% ethyl cellulose and atorvastatin.

## 3. Results and Discussion

### 3.1. Preparation of the Electrospun Nanofibers

In electrospinning, the capacity of the droplet to acquire charge is directly influenced by the solution’s features, like the electrical conductivity and dielectric constant, which affect the formation of numerous jets [55]. In this study, FA with a dielectric-constant value of 58 was used with a concentration of 10% *w*/*v*. This concentration was appropriate to achieve enough electrical conductivity for electrospinning without significantly reducing the solution’s viscosity over time, as was observed for solutions with greater FA contents (20% and higher) [56]. It is worth mentioning that the AA dielectric constant is only 6.2 [57]. Therefore, acetic and formic acids (AA/FA) were used with a ratio of 9:1 *w*/*w* in this study.

### 3.2. Characterization of the Prepared Nanofibers

#### 3.2.1. Viscosity of the Spinning Solutions

Although electrospinning is an uncomplicated process, numerous parameters need to be adjusted to obtain the necessary properties from the resulting nanofiber membranes. One of the primary factors considered is the electrospun solution’s viscosity. Too much or too little viscosity lead to an imbalance between the electrostatic force and viscous solution force required for homogeneous fiber development, resulting in bead formation or beads on fibers [58].

In electrospinning, a thin liquid jet is expelled from the droplet when the electric field exceeds the droplet’s surface tension. Charge repulsion and viscoelastic forces compete to regulate the behavior of the jet. Stable electrospinning is possible above some critical solution concentrations, allowing entanglements of molecules to obtain adequate viscoelasticity of the solution. Due to liquid–surface tension, insufficient chain entanglement (low viscoelasticity) causes the formation of a spray instead of a continuous jet [59].

In this study, SBG was incorporated in concentrations of 3, 5, 10 and 20% *w*/*w* into C1 to produce C1-B3, C1-B5, C1-B10 and C1-B20, respectively (Table 1). It is obvious from the viscosity data for the electrospun preparations that as the amount of bioactive glass increases, the viscosity of the solution drops, most likely because of the drop in PCL concentration. Thus, it can be found from the rheology investigation that C1-B20 has the lowest viscosity of all the employed solutions. When using a low-viscosity electrospinning solution, no fibers are formed due to the breakup of the electrospun jet before it reaches the collector due to high surface tension and low viscoelastic forces [60,61]. Furthermore, the increase in the amount of insoluble solids suspended in the solution leads to electrospraying rather than electrospinning [62]. Therefore, electrospinning of solutions with higher SBG content was performed at a higher voltage (Table 1) to form the fibers. 

#### 3.2.2. Characterization of Nanofibers

##### Scanning Electron Microscopy (SEM)

**Figure 2:**
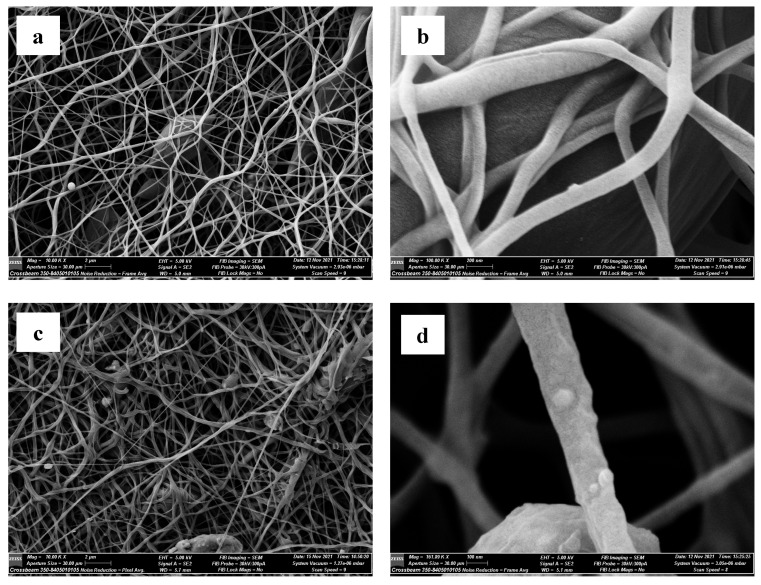
SEM images for C1 fibers prepared without SBG (**a**,**b**) and prepared with SBG (**c**,**d**).

The SEM micrographs of C1 nanofibers demonstrated uniform and highly smooth nanofibers with an average fiber diameter of 118.18 ± 21.91 nm and practically random architecture (Figure 2a,b). The nanofiber membrane showed an interconnecting porous structure that is key to enhancing cell proliferation [63]. Nanofibers prepared without SBG showed gap distance values between the fibers ranging from 1.37 to 2.04 µm, and a comparative range between 1.41 and 2.26 µm was found for nanofibers prepared with SBG. Nanofibers prepared with SBG (Figure 2c,d) showed SBG particles on their surface as well as some bead defects, which may be because SBG nanoparticles retard the ability of ejected solution droplets to be stretched and whipped in the air to form a fiber. Furthermore, this can be attributed to the low viscosity of solutions containing SBG and its negative effect on the electrospinning process, as discussed in the previous section. 

##### Fourier Transform Infrared (FT-IR) Spectra and X-ray Diffraction (XRD) Analysis

**Figure 3:**
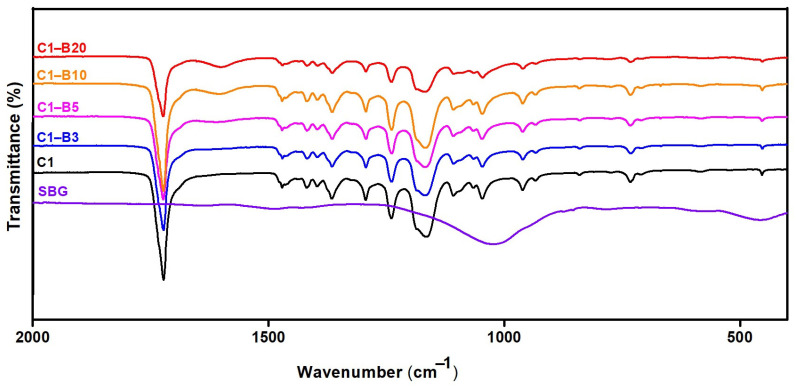
FT-IR spectra for PCL fibers prepared without SBG (C1) and with various SBG amounts.

FT-IR analysis (Figure 3) clearly indicates several vibrations from PCL. Based on the FTIR spectrum for pure PCL fibers (C1), it was evident that the most intense PCL vibration was the carbonyl group of the ester group at 1720 cm^−1^. The C-O-H-bent band (carboxylic acid) was shown at 1448 cm^−1^ in-plane and 988 cm^−1^ out-of-plane. At 1300–1000 cm^−1^, significant C-O stretching bands were observed. A characteristic band at 729 cm^−1^ corresponded to the methylene groups in the bending mode of a scissor-like fashion [64,65,66]. In the case of SBG, there were two distinguishing peaks; one was located at 1038 cm^−1^ and is ascribed to P-O stretching vibrations, and the other was located at 933 cm^−1^ and is related to the Si-O-Si stretching of non-bridging oxygen atoms. Other peaks could be observed at 731 cm^−1^ and 459 cm^−1^, which are attributed to the Si-O-Si symmetric stretch of oxygen atoms bridging between tetrahedrals and the P-O bending vibration, respectively [48]. The FT-IR spectra of PCL did not show any essential differences after the addition of SBG. A new band was found at 1600 cm^−1^ for the PCL-SBG materials that was not observed in the C1 or pure SBG spectra. It is evident that the intensity of this new band at 1600 cm^−1^ increased with SBG content. It cannot be excluded that this new band in PCL-SBG fibers is due to specific interactions between PCL and SBG and the possible formation of molecular bonds. A thorough explanation of the nature of such bonds requires an in-depth analysis.

**Figure 4:**
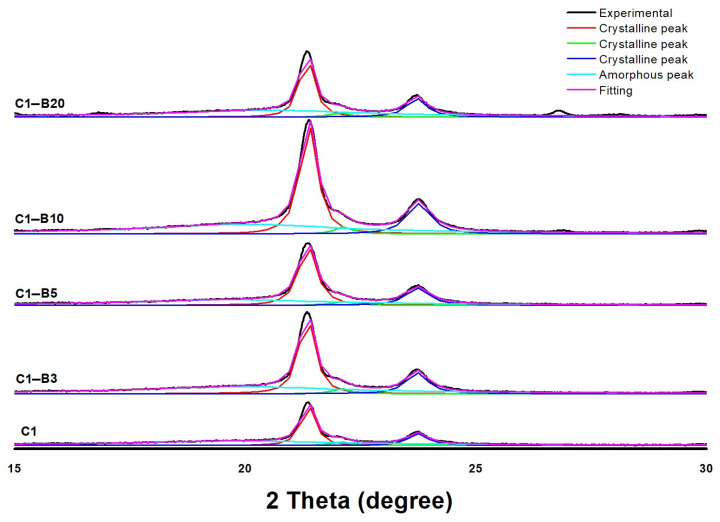
X-ray diffractions for PCL fibers prepared without SBG (C1) and prepared with SBG.

In the case of the XRD results (Figure 4), there were three PCL diffraction peaks in the range of 2θ between 10 and 33 deg in addition to the broad, amorphous halo. The diffraction peaks were located at 2θ of around 21.3°, 22.05° and 23.7°, with slight shifts among samples. According to deconvolution results, the peaks represented diffraction from the (110), (111) and (200) crystallographic planes. The position of the maximum amorphous halo was around 19.90° [23,67]. PCL samples containing SBG indicated peaks from SBG in addition to PCL peaks, with the intensity increasing with the SBG content, suggesting the existence of a crystalline structure in SBG. However, it was only clearly visible for samples with 10% and 20% SBG content. The measurements of XRD crystallinity (Table 2) performed using all analyzed and deconvoluted peaks clearly indicated an increase in crystallinity by ca. 20% (relatively) from the value 0.37 for pure PCL nanofibers to about 0.45 after the addition of SBG (averaged from the 0.43–0.48 range of results). This observation suggests an additional crystallization induced by nanoparticles of SBG, which may suggest heterogeneous crystallization. That mechanism may be supported by high supersaturation during fast solvent evaporation. 

##### Mechanical Tests

**Figure 5:**
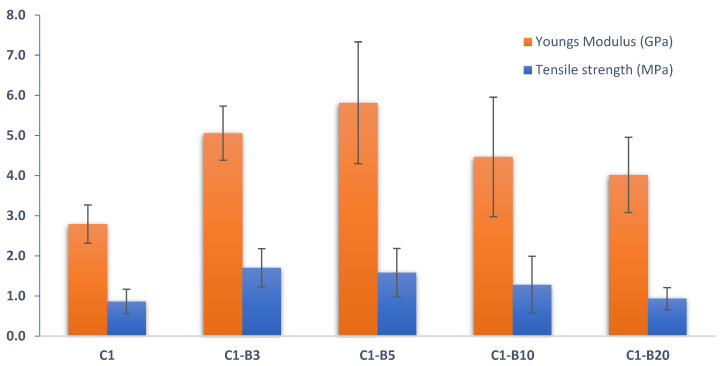
Mechanical properties of PCL-based nanofibers.

Tensile strength and Young’s modulus were evaluated from the stress–strain diagrams of the PCL-based nanofiber membranes. Our results (Figure 5) indicated a quite complex effect of the SBG component on the mechanical characteristics of the nanofibers. There was a significant (*p* < 0.05) enhancement in Young’s modulus and tensile strength of membranes with 3 and 5% SBG content, followed by a reduction in mechanical properties at higher SBG concentrations. The initial growth of mechanical properties can be attributed to the stiffness of SBG, which amplified the fiber’s strength [68]. The reduction in both mechanical parameters at higher SBG content is most probably related to SBG particle clustering, which causes each component to work as a rigid constituent and prevents crazes from moving during tensile loading [69].

The PCL nanofiber membrane with 5% bioactive glass (C1-B5) was chosen for additional research due to its superior mechanical qualities. C1-B5 nanofibers were loaded with atorvastatin with and without ethyl cellulose as a coating polymer. Then the nanofibers were tested for drug release and for their influence on human skin fibroblast cell lines’ ability to proliferate.

#### 3.2.3. Loading Atorvastatin on the Nanofibers and In Vitro Drug Release

**Figure 6:**
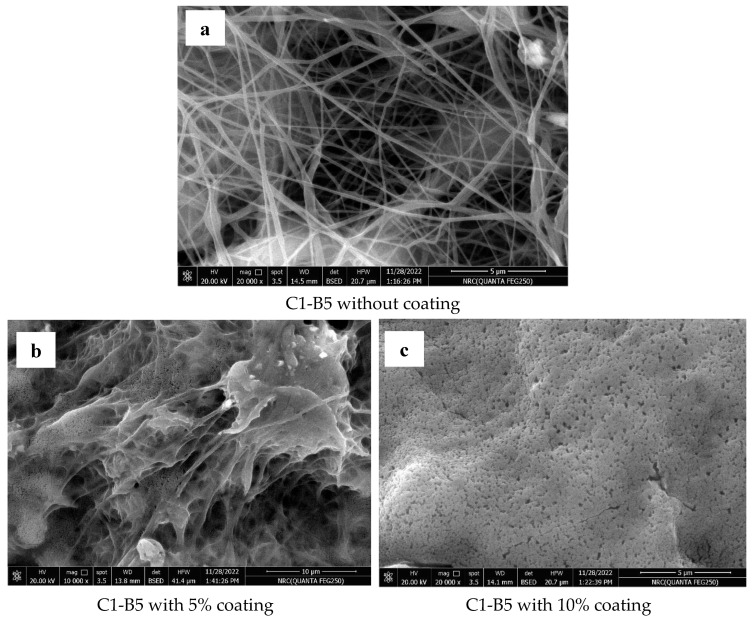

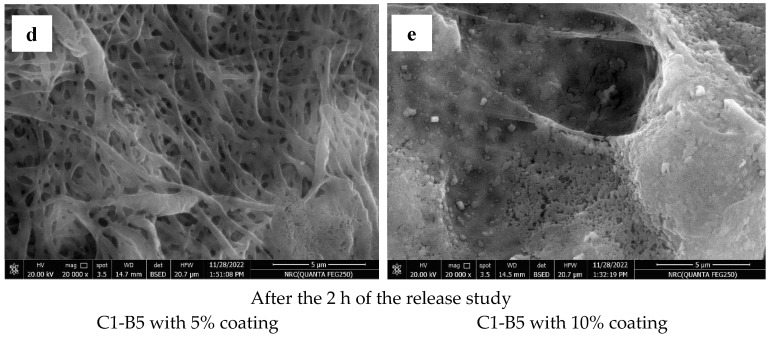
(**a**–**e**) SEM images of C1-B5 nanofiber membranes with and without coating with ethyl cellulose (5 and 10% *w*/*v* coating solution).

The selected nanofibers (C1-B5) were coated with ethyl cellulose using the dip-coating technique. It was found that the ethyl cellulose deposed onto the PCL fibers’ structure (Figure 6b,c). According to previous studies, the connection between ethyl cellulose and PCL may be attributed to potential hydrogen bonding between the OH group in ethyl cellulose and the COOH end groups of PCL [70].

**Figure 7:**
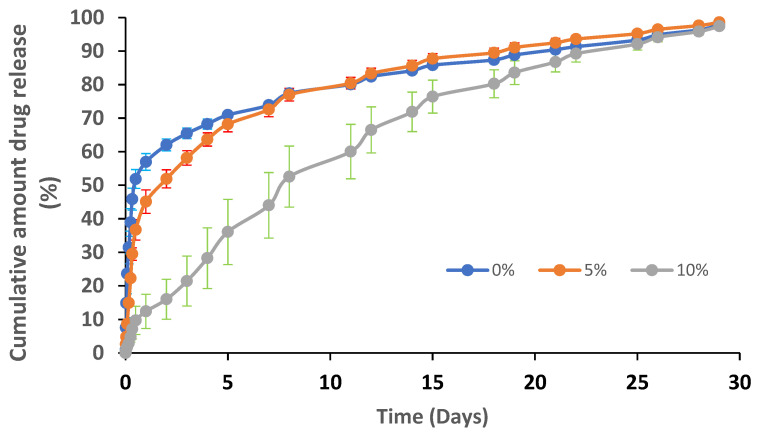
In vitro drug release profiles of atorvastatin through C1-B5 nanofibers using 0, 5 and 10% of the coating polymer.

Figure 7 reveals the release profiles of atorvastatin from the nanofibers C1-B5 prepared with/without the use of a coating layer of ethyl cellulose. Drug release from the tested samples was calculated in percentage depending on their drug loading values, which were 2.5 mg/cm^2^ for the nanofibers coating the polymer (5 and 10%) and 1.5 mg/cm^2^ for the non-coated nanofibers.

The nanofibers coated with ethyl cellulose revealed a significant change in their release profiles compared to the same nanofibers prepared without the coating polymer. The release mechanism of atorvastatin from the coated polymers was found to be a non-Fickian diffusion pattern (diffusional exponent values “n” equal to 0.49 and 0.75 for nanofibers coated with 5% and 10% ethyl cellulose, respectively). Consequently, the release of atorvastatin from these nanofibers depended on polymer erosion and diffusion. On the other hand, the “n” value for nanofiber prepared without coating equaled 0.35, where the release of atorvastatin was likely to be Fickian-diffusion driven. 

Nanofibers coated with 10% polymer demonstrated the highest mean release time (MRT) value (9.57 days) and the lowest release efficiency (RE) value (65.44%), followed by the nanofibers coated with 5% polymer (MRT = 5.19 days, and RE = 81.11%) and finally the nanofibers prepared without the coating layer (MRT = 4.75 days, and RE = 81.73%). The release profile for the formulation coated with 10% ethyl cellulose showed gradual drug release without initial burst release, as compared to the non-coated fibers and the coated fibers with 5% ethyl cellulose. As shown in the SEM images in Figure 6, increasing the concentration of the coating polymer formed a more compact layer in the spaces between the nanofibers, resulting in a retardation of the drug release through the tightened areas between the nanofibers, where the average spaces between nanofibers containing 10%, 5%, and 0% of coating polymer equal 0.625 µm, 1.2 µm and 1.74 µm, respectively (Figure 6c, Figure 6b and Figure 6a, respectively). Furthermore, the degradation of the 10% ethyl cellulose in the nanofiber membrane (Figure 7e) was slower than that for the 5% ethyl cellulose (Figure 6d) where, after 2 h of the release study, the porous structure in the nanofibers coated with 5% ethyl cellulose was more visible, and this was the reason why the release profiles for the uncoated and 5% coated nanofibers were similar after 5 h of the release study.

#### 3.2.4. Ex Vivo Studies on the Human Skin Fibroblast Cell Line

It is well known that wound healing is a dynamic process that involves various stages [71]. Hemostasis is the first stage of wound healing, followed by the inflammatory, proliferative, and maturation stages. 

##### Cytotoxicity Assay (MTT Assay)

**Figure 8:**
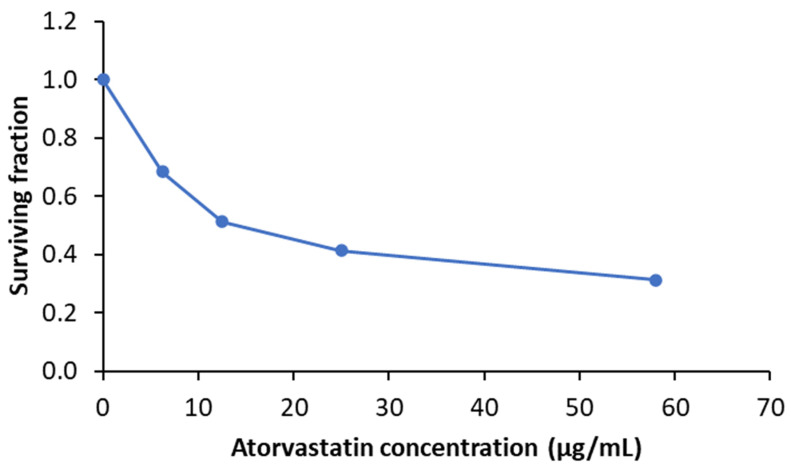
The cytotoxic effect of atorvastatin on human skin fibroblast cell line that was measured by MTT assay.

In our study, an MTT assay was utilized to determine the cytotoxicity of atorvastatin on the human skin fibroblast cell line. The surviving fractions (cell viability) for the human skin fibroblast cell line were decreased in a dose-dependent manner (Figure 8), and the results revealed that the IC50 value (50% inhibitory concentration) for atorvastatin was 13.5 μg/mL. The nanofibers used in the wound healing assay were loaded with a drug concentration equivalent to 10% of the IC50 concentration. 

##### Wound Healing Assay

The wound healing test is a common in vitro method for determining the migration of cells. Furthermore, obtaining quick and effective wound closure is the main objective of wound treatment. The wound healing assay is simple to use for small molecule screening and drug discovery [72,73].

**Figure 9:**
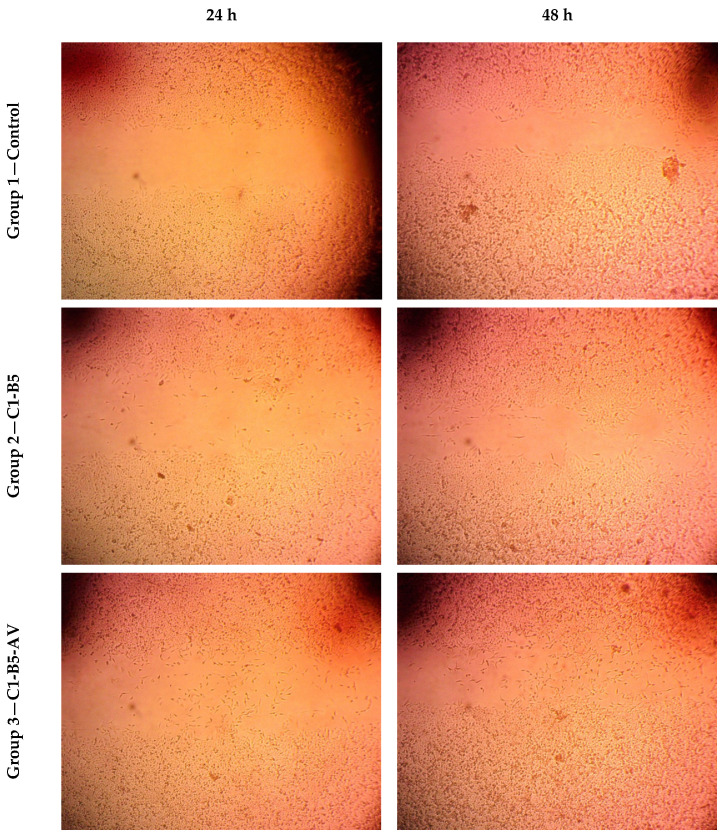

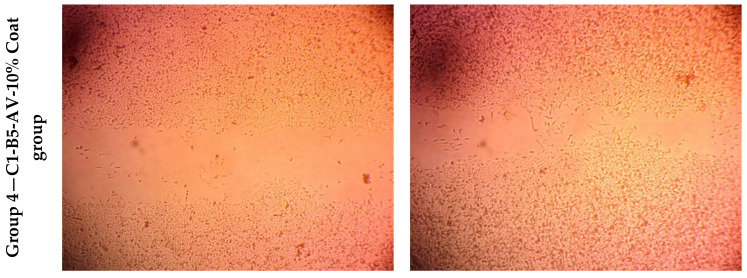
The scratch wound test in the HSF cell line shows the progression in healing after 24 and 48 h of treatment with different nanofiber membranes (magnification × 40).

Figure 9 illustrates how the HSF cell line’s wound sizes changed after 24 h and 48 h after treatment with different nanofiber membranes. Nanofiber membranes loaded with a bioactive glass (C1-B5 group) accelerated wound closure within 24 h of the study compared to the same formulation prepared without the bioactive glass (Cl group), as shown from the values for the gap distance between the cells (0.31 ± 0.04 and 0.53 ± 0.04 mm, respectively). This highlights the effect of bioactive glass on enhancing fibroblast migration and proliferation [45] by overexpressing some important growth factors in the cells [41].

To examine the effect of atorvastatin in this study, the results for the C1-B5-AV group and the C1-B5-AV-10% coated group were examined. These two groups showed similar pronounced promotion of wound healing after 24 h (0.19 ± 0.02 and 0.18 ±0.06 mm, respectively) and 48 h (0.14 ± 0.04 and 0.13 ± 0.01 mm, respectively), which suggests that atorvastatin can promote wound healing (*p* < 0.05 compared to the other tested groups). Furthermore, in these two groups, cells were progressively motile and filled the free edge (scratch) after 48 h. Such a reconstruction role for atorvastatin is due to the ability of statins to increase dermal fibroblast proliferation [74], as they have the ability to modulate the inflammatory response by increasing the number of keratinocytes in the skin [75].

In conclusion, our tested nanofibers, prepared using PCL and containing bioactive glass and atorvastatin, significantly promoted wound healing in a human skin fibroblast cell line. Such formulations open new avenues for the treatment of wounds and advance modern medicine because the market availability of existing topical wound healing medications is limited.

## 4. Conclusions

Wound dressings as nanofiber membranes are essential to treating wounds. In our study, PCL-based nanofiber membranes were prepared using electrospinning technology. SBGs were combined with the nanofibers in different concentrations. The maximum mechanical properties were achieved at 5% of SBGs. The nanofiber membranes showed a porous structure with a mean fiber diameter of 118.18 ± 21.91 mm. The results indicated that SBG nanoparticles induce additional crystallization of PCL. Coating nanofiber membranes using ethyl cellulose (5 and 10% *w*/*v*) resulted in retardation of atorvastatin release. Nanofiber membranes with bioactive glass and atorvastatin promoted the healing of human skin fibroblast cell lines. In conclusion, the synergistic effect of the nanofiber membrane structure and each bioactive glass and atorvastatin on wound healing can highlight the effectiveness of using such a combination in wound dressing.

## Figures and Tables

**Table 1:** Composition and viscosity of the prepared spinning polymeric solutions and the applied voltage in the electrospinning process.

	Spinning Polymeric Solution			Applied Voltage (kV)
Sample Code	Solid Content Composition (% *w*/*w*) *		Viscosity (mPas) ± SD	
	PCL	SBG		
C1	10.0	0.0	507.86 ± 6.79	15
C1-B3	9.7	0.3	491.43 ± 5.92	17
C1-B5	9.5	0.5	489.13 ± 7.85	19
C1-B10	9.0	1.0	478.30 ± 10.80	21
C1-B20	8.0	2.0	337.30 ± 7.41	25

* The solid content in the nanofibers represented 10% *w*/*w*, and the solvent used (90%) was a blend of acetic acid and formic acid (AA/FA) with a ratio of 9:1 *w*/*w*.

**Table 2:** XRD crystallinity for the investigated samples.

Sample Code	PCL Crystallinity
C1	0.37
C1-B3	0.44
C1-B5	0.47
C1-B10	0.48
C1-B20	0.43

## Data Availability

The data presented in this study are available on request from the corresponding author.

## References

[1] Ather S., Harding K., Tate S., Rajendran S. (2019). Wound management and dressings. Advanced Textiles for Wound Care.

[2] Armstrong D.G., Wrobel J., Robbins J.M. (2007). Guest Editorial: Are diabetes-related wounds and amputations worse than cancer?. Int. Wound J..

[3] Richmond N.A., Lamel S.A., Davidson J.M., Martins-Green M., Sen C.K., Tomic-Canic M., Vivas A.C., Braun L.R., Kirsner R.S. (2013). US-National Institutes of Health-funded research for cutaneous wounds in 2012. Wound Repair Regen..

[4] Wallace H.A., Basehore B.M., Zito P.M. (2023). Wound Healing Phases. StatPearls.

[5] Van Koppen C.J., Hartmann R.W. (2015). Advances in the treatment of chronic wounds: A patent review. Expert Opin. Ther. Pat..

[6] Qin W., Li J., Tu J., Yang H., Chen Q., Liu H. (2017). Fabrication of porous chitosan membranes composed of nanofibers by low temperature thermally induced phase separation, and their adsorption behavior for Cu^2+^. Carbohydr. Polym..

[7] Kamin Z., Abdulrahim N., Misson M., Chiam C., Sarbatly R., Krishnaiah D., Bono A. (2021). Use of melt blown polypropylene nanofiber templates to obtain homogenous pore channels in glycidyl methacrylate/ethyl dimethacrylate-based monoliths. Chem. Eng. Commun..

[8] MacNeil S. (2008). Biomaterials for tissue engineering of skin. Mater. Today.

[9] Kanungo I., Fathima N.N., Rao J.R., Nair B.U. (2013). Influence of PCL on the material properties of collagen based biocomposites and in vitro evaluation of drug release. Mater. Sci. Eng. C.

[10] Liu X., Xu H., Zhang M., Yu D.G. (2021). Electrospun Medicated Nanofibers for Wound Healing: Review. Membranes.

[11] Rafiq M., Rather S.-u., Wani T.U., Rather A.H., Khan R.S., Khan A.E., Hamid I., Khan H.A., Alhomida A.S., Sheikh F.A. (2023). Recent progress in MXenes incorporated into electrospun nanofibers for biomedical application: Study focusing from 2017 to 2022. Chin. Chem. Lett..

[12] Xue J., Xie J., Liu W., Xia Y. (2017). Electrospun Nanofibers: New Concepts, Materials, and Applications. Acc. Chem. Res..

[13] Ramakrishna S., Fujihara K., Teo W.-E., Yong T., Ma Z., Ramaseshan R. (2006). Electrospun nanofibers: Solving global issues. Mater. Today.

[14] Xue J., Wu T., Dai Y., Xia Y. (2019). Electrospinning and Electrospun Nanofibers: Methods, Materials, and Applications. Chem. Rev..

[15] Toriello M., Afsari M., Shon H.K., Tijing L.D. (2020). Progress on the Fabrication and Application of Electrospun Nanofiber Composites. Membranes.

[16] Akhmetova A., Heinz A. (2020). Electrospinning Proteins for Wound Healing Purposes: Opportunities and Challenges. Pharmaceutics.

[17] Amajuoyi J.N., Ilomuanya M.O., Asantewaa-Osei Y., Azubuike C.P., Adeosun S.O., Igwilo C.I. (2020). Development of electrospun keratin/coenzyme Q10/poly vinyl alcohol nanofibrous scaffold containing mupirocin as potential dressing for infected wounds. Future J. Pharm. Sci..

[18] Labet M., Thielemans W. (2009). Synthesis of polycaprolactone: A review. Chem. Soc. Rev..

[19] Ceonzo K., Gaynor A., Shaffer L., Kojima K., Vacanti C.A., Stahl G.L. (2006). Polyglycolic acid-induced inflammation: Role of hydrolysis and resulting complement activation. Tissue Eng..

[20] Bhullar S.K., Rana D., Lekesiz H., Bedeloglu A.C., Ko J., Cho Y., Aytac Z., Uyar T., Jun M., Ramalingam M. (2017). Design and fabrication of auxetic PCL nanofiber membranes for biomedical applications. Mater. Sci. Eng. C Mater. Biol. Appl..

[21] Ma K., Liao C., Huang L., Liang R., Zhao J., Zheng L., Su W. (2021). Electrospun PCL/MoS(2) Nanofiber Membranes Combined with NIR-Triggered Photothermal Therapy to Accelerate Bone Regeneration. Small.

[22] Xue J., He M., Liang Y., Crawford A., Coates P., Chen D., Shi R., Zhang L. (2014). Fabrication and evaluation of electrospun PCL-gelatin micro-/nanofiber membranes for anti-infective GTR implants. J. Mater. Chem. B.

[23] Ravichandran S., Radhakrishnan J. (2022). Anticancer efficacy of lupeol incorporated electrospun Polycaprolactone/gelatin nanocomposite nanofibrous mats. Nanotechnology.

[24] Haroosh H.J., Dong Y., Jasim S., Ramakrishna S. (2022). Morphological Structures and Drug Release Effect of Multiple Electrospun Nanofibre Membrane Systems Based on PLA, PCL, and PCL/Magnetic Nanoparticle Composites. J. Nanomater..

[25] McIver L.A., Siddique M.S. (2023). Atorvastatin. StatPearls.

[26] Lipitor (2009). Atorvastatin Calcium Tablets: Description.

[27] Suzuki-Banhesse V.F., Azevedo F.F., Araujo E.P., do Amaral M.E., Caricilli A.M., Saad M.J., Lima M.H. (2015). Effect of atorvastatin on wound healing in rats. Biol. Res. Nurs..

[28] Zhou Q., Liao J.K. (2010). Pleiotropic effects of statins—Basic research and clinical perspectives. Circ. J..

[29] Choudhury H., Gorain B., Pandey M., Chatterjee L.A., Sengupta P., Das A., Molugulu N., Kesharwani P. (2017). Recent update on nanoemulgel as topical drug delivery system. J. Pharm. Sci..

[30] Farsaei S., Khalili H., Farboud E.S. (2012). Potential role of statins on wound healing: Review of the literature. Int. Wound J..

[31] Tejaswini T., Keerthana M., Vidyavathi M., Kumar R. (2020). Design and evaluation of atorvastatin-loaded chitosan-hydroxyapatite composite bioscaffolds for wound-healing activity. Future J. Pharm. Sci..

[32] Mostafa A.A., Zaazou M.H., Chow L.C., Mahmoud A.A., Zaki D.Y., Basha M., Hamid M.A.A., Khallaf M.E., Sharaf N.F., Hamdy T.M. (2015). Injectable nanoamorphous calcium phosphate based in situ gel systems for the treatment of periapical lesions. Biomed. Mater..

[33] Morsy M.A., Abdel-Latif R.G., Nair A.B., Venugopala K.N., Ahmed A.F., Elsewedy H.S., Shehata T.M. (2019). Preparation and Evaluation of Atorvastatin-Loaded Nanoemulgel on Wound-Healing Efficacy. Pharmaceutics.

[34] Abootorabi S., Akbari J., Saeedi M., Seyedabadi M., Ranaee M., Asare-Addo K., Nokhodchi A. (2022). Atorvastatin Entrapped Noisome (Atrosome): Green Preparation Approach for Wound Healing. AAPS PharmSciTech.

[35] Zeng W., Cheng N.M., Liang X., Hu H., Luo F., Jin J., Li Y.W. (2022). Electrospun polycaprolactone nanofibrous membranes loaded with baicalin for antibacterial wound dressing. Sci. Rep..

[36] Naseri S., Lepry W.C., Nazhat S.N. (2017). Bioactive glasses in wound healing: Hope or hype?. J. Mater. Chem. B.

[37] Ma J., Wu C. (2022). Bioactive inorganic particles-based biomaterials for skin tissue engineering. Exploration.

[38] Miguez-Pacheco V., Hench L.L., Boccaccini A.R. (2015). Bioactive glasses beyond bone and teeth: Emerging applications in contact with soft tissues. Acta Biomater..

[39] Day R.M. (2005). Bioactive glass stimulates the secretion of angiogenic growth factors and angiogenesis in vitro. Tissue Eng..

[40] Day R.M., Boccaccini A.R. (2005). Effect of particulate bioactive glasses on human macrophages and monocytes in vitro. J. Biomed. Mater. Res. Part A.

[41] Li H., He J., Yu H., Green C.R., Chang J. (2016). Bioglass promotes wound healing by affecting gap junction connexin 43 mediated endothelial cell behavior. Biomaterials.

[42] Stoor P., Soderling E., Salonen J.I. (1998). Antibacterial effects of a bioactive glass paste on oral microorganisms. Acta Odontol. Scand..

[43] Reffitt D.M., Ogston N., Jugdaohsingh R., Cheung H.F., Evans B.A., Thompson R.P., Powell J.J., Hampson G.N. (2003). Orthosilicic acid stimulates collagen type 1 synthesis and osteoblastic differentiation in human osteoblast-like cells in vitro. Bone.

[44] Finnson K.W., McLean S., Di Guglielmo G.M., Philip A. (2013). Dynamics of Transforming Growth Factor Beta Signaling in Wound Healing and Scarring. Adv. Wound Care New Rochelle.

[45] Xie W., Chen X., Miao G., Tang J., Fu X. (2016). Regulation of cellular behaviors of fibroblasts related to wound healing by sol-gel derived bioactive glass particles. J. Biomed. Mater. Res. A.

[46] Elshazly N., Saad M.M., El Backly R.M., Hamdy A., Patruno M., Nouh S., Saha S., Chakraborty J., Marei M.K. (2023). Nanoscale borosilicate bioactive glass for regenerative therapy of full-thickness skin defects in rabbit animal model. Front. Bioeng. Biotechnol..

[47] Wang Y., Li T., Xie C., Li S., Lei B. (2023). Multi-layer-structured bioactive glass nanopowder for multistage-stimulated hemostasis and wound repair. Bioact. Mater..

[48] El-Sayed M.M., Mostafa A.A., Gaafar A.M., El Hotaby W., Hamzawy E.M., El-Okaily M.S., Gamal-Eldeen A.M. (2017). In vitro kinetic investigations on the bioactivity and cytocompatibility of bioactive glasses prepared via melting and sol-gel techniques for bone regeneration applications. Biomed. Mater..

[49] Mabrouk M., Mostafa A., Oudadesse H., Wers E., Lucas-Girot A., El-Gohary M.I. (2014). Comparative study of nanobioactive glass quaternary system 46S6. Bioceram. Dev. Appl..

[50] Mendyk A., Jachowicz R., Fijorek K., Dorożyński P., Kulinowski P., Polak S. (2012). KinetDS: An open source software for dissolution test data analysis. Dissolution Technol..

[51] Podczeck F. (1993). Comparison of in vitro dissolution profiles by calculating mean dissolution time (MDT) or mean residence time (MRT). Int. J. Pharm..

[52] Korsmeyer R.W., Gurny R., Doelker E., Buri P., Peppas N.A. (1983). Mechanisms of solute release from porous hydrophilic polymers. Int. J. Pharm..

[53] Peppas N. (1985). Analysis of Fickian and non-Fickian drug release from polymers. Pharm. Acta Helv..

[54] Liang C.C., Park A.Y., Guan J.L. (2007). In vitro scratch assay: A convenient and inexpensive method for analysis of cell migration in vitro. Nat. Protoc..

[55] Wu Y.K., Wang L., Fan J., Shou W., Zhou B.M., Liu Y. (2018). Multi-Jet Electrospinning with Auxiliary Electrode: The Influence of Solution Properties. Polymers.

[56] Denis P., Dulnik J., Sajkiewicz P. (2015). Electrospinning and structure of bicomponent polycaprolactone/gelatin nanofibers obtained using alternative solvent system. Int. J. Polym. Mater. Polym. Biomater..

[57] Luo C., Stride E., Edirisinghe M. (2012). Mapping the influence of solubility and dielectric constant on electrospinning polycaprolactone solutions. Macromolecules.

[58] Nezarati R.M., Eifert M.B., Cosgriff-Hernandez E. (2013). Effects of humidity and solution viscosity on electrospun fiber morphology. Tissue Eng. Part C Methods.

[59] Han D., Steckl A.J. (2019). Coaxial electrospinning formation of complex polymer fibers and their applications. ChemPlusChem.

[60] Pillay V., Dott C., Choonara Y.E., Tyagi C., Tomar L., Kumar P., du Toit L.C., Ndesendo V.M. (2013). A review of the effect of processing variables on the fabrication of electrospun nanofibers for drug delivery applications. J. Nanomater..

[61] Medeiros G.B., Lima F.d.A., de Almeida D.S., Guerra V.G., Aguiar M.L. (2022). Modification and Functionalization of Fibers Formed by Electrospinning: A Review. Membranes.

[62] El-Okaily M.S., El-Rafei A.M., Basha M., Abdel Ghani N.T., El-Sayed M.M.H., Bhaumik A., Mostafa A.A. (2021). Efficient drug delivery vehicles of environmentally benign nano-fibers comprising bioactive glass/chitosan/polyvinyl alcohol composites. Int. J. Biol. Macromol..

[63] Xin X., Hussain M., Mao J.J. (2007). Continuing differentiation of human mesenchymal stem cells and induced chondrogenic and osteogenic lineages in electrospun PLGA nanofiber scaffold. Biomaterials.

[64] Elzein T., Nasser-Eddine M., Delaite C., Bistac S., Dumas P. (2004). FTIR study of polycaprolactone chain organization at interfaces. J. Colloid Interface Sci..

[65] Ramirez-Cedillo E., Ortega-Lara W., Rocha-Pizana M.R., Gutierrez-Uribe J.A., Elias-Zuniga A., Rodriguez C.A. (2019). Electrospun Polycaprolactone Fibrous Membranes Containing Ag, TiO(2) and Na(2)Ti(6)O(13) Particles for Potential Use in Bone Regeneration. Membranes.

[66] Alahmmar M., Prabhakaran P., Jaganathan S., Nik N.A.N. (2023). Fabrication and Characterization of Polycaprolactone with Retinoic Acid and Cerium Oxide for Anticancer Applications. Biointerface Res. Appl. Chem..

[67] Kołbuk D., Sajkiewicz P., Maniura-Weber K., Fortunato G. (2013). Structure and morphology of electrospun polycaprolactone/gelatine nanofibres. Eur. Polym. J..

[68] Otadi M., Mohebbi-Kalhori D. (2015). Evaluate of different bioactive glass on mechanical properties of nanocomposites prepared using electrospinning method. Procedia Mater. Sci..

[69] Balaji S., Kumar R., Sripriya R., Kakkar P., Ramesh D.V., Reddy P.N.K., Sehgal P. (2012). Preparation and comparative characterization of keratin–chitosan and keratin–gelatin composite scaffolds for tissue engineering applications. Mater. Sci. Eng. C.

[70] Beikzadeh S., Hosseini S.M., Mofid V., Ramezani S., Ghorbani M., Ehsani A., Mortazavian A.M. (2021). Electrospun ethyl cellulose/poly caprolactone/gelatin nanofibers: The investigation of mechanical, antioxidant, and antifungal properties for food packaging. Int. J. Biol. Macromol..

[71] Lopez-Jornet P., Camacho-Alonso F., Gomez-Garcia F., Molina Minano F., Canas X., Serafin A., Castillo J., Vicente-Ortega V. (2014). Effects of potassium apigenin and verbena extract on the wound healing process of SKH-1 mouse skin. Int. Wound J..

[72] Suntar I., Kupeli Akkol E., Keles H., Yesilada E., Sarker S.D. (2013). Exploration of the wound healing potential of Helichrysum graveolens (Bieb.) Sweet: Isolation of apigenin as an active component. J. Ethnopharmacol..

[73] Hulkower K.I., Herber R.L. (2011). Cell migration and invasion assays as tools for drug discovery. Pharmaceutics.

[74] Maged A., Abdelkhalek A.A., Mahmoud A.A., Salah S., Ammar M.M., Ghorab M.M. (2019). Mesenchymal stem cells associated with chitosan scaffolds loaded with rosuvastatin to improve wound healing. Eur. J. Pharm. Sci..

[75] Tomic-Canic M. (2012). The Role of Statins in Cutaneous Wound Healing.

